# Activation of a hypothalamus-habenula circuit by mechanical stimulation inhibits cocaine addiction-like behaviors

**DOI:** 10.1186/s40659-023-00440-7

**Published:** 2023-05-17

**Authors:** Han Byeol Jang, DanBi Ahn, Suchan Chang, Hyung Kyu Kim, Bong Hyo Lee, Sang Chan Kim, Scott C. Steffensen, Kyle B. Bills, Hubert Lee, Hee Young Kim

**Affiliations:** 1 Department of Physiology, Yonsei University College of Medicine, Seoul, 03722 South Korea; 2 Department of Physiology, College of Korean Medicine, Daegu Haany University, Daegu, 42158 South Korea; 3 Medical Research Center, College of Korean Medicine, Daegu Haany University, Gyeongsan, 38610 South Korea; 4 Department of Psychology and Neuroscience, Brigham Young University, Provo, UT 84602 USA; 5 Department of Biomedical Sciences, Noorda College of Osteopathic Medicine, Provo, UT 84606 USA; 6 Department of Pharmacology and Toxicology, University of Texas Medical Branch, 301 University Boulevard, Galveston, TX 77555 USA

**Keywords:** Mechanical stimulation (MS), Cocaine, Lateral habenula, Locomotor activity, Ultrasonic vocalization (USVs), Optogenetics

## Abstract

**Background:**

Mechanoreceptor activation modulates GABA neuron firing and dopamine (DA) release in the mesolimbic DA system, an area implicated in reward and substance abuse. The lateral habenula (LHb), the lateral hypothalamus (LH), and the mesolimbic DA system are not only reciprocally connected, but also involved in drug reward. We explored the effects of mechanical stimulation (MS) on cocaine addiction-like behaviors and the role of the LH-LHb circuit in the MS effects. MS was performed over ulnar nerve and the effects were evaluated by using drug seeking behaviors, optogenetics, chemogenetics, electrophysiology and immunohistochemistry.

**Results:**

Mechanical stimulation attenuated locomotor activity in a nerve-dependent manner and 50-kHz ultrasonic vocalizations (USVs) and DA release in nucleus accumbens (NAc) following cocaine injection. The MS effects were ablated by electrolytic lesion or optogenetic inhibition of LHb. Optogenetic activation of LHb suppressed cocaine-enhanced 50 kHz USVs and locomotion. MS reversed cocaine suppression of neuronal activity of LHb. MS also inhibited cocaine-primed reinstatement of drug-seeking behavior, which was blocked by chemogenetic inhibition of an LH-LHb circuit.

**Conclusion:**

These findings suggest that peripheral mechanical stimulation activates LH-LHb pathways to attenuate cocaine-induced psychomotor responses and seeking behaviors.

**Supplementary Information:**

The online version contains supplementary material available at 10.1186/s40659-023-00440-7.

## Background

There is growing evidence that stimulation of peripheral nerve modulates substrates in the supraspinal central nervous system (CNS) outside the somatosensory circuits [1, 2]. Our previous studies have found that mechanical stimulation applied to the peripheral nerve modulates GABA neurons in ventral tegmental area (VTA) and dopamine (DA) release in the mesolimbic DA system, an area implicated in reward and motivation [2, 3]. Our animal studies also revealed that mechanical stimulation of ulnar nerve suppresses the hyper-locomotor activity induced by cocaine administration and these effects are mediated by activation of A-fibers at the nerve trunk [4] and the dorsal column-medial lemniscus pathway [3, 5]. However, the use of mechanoreceptor-based therapies in the treatment of substance abuse and the underlying mechanisms remain largely unexplored.

The lateral hypothalamus (LH) is known to play a crucial role in regulating feeding and reward-related behaviors [6]. It contains distinct glutamatergic and GABAergic subpopulations with glutamatergic projections from the LH innervating the lateral habenula (LHb) [7, 8]. These projections, in turn, project directly to VTA neurons [8]. Furthermore, the LHb plays an important role in processing aversive signals that contribute to reward function and addiction [9]. Stimulation of the LHb inhibits VTA neurons to reduce NAc DA release [9, 10].

To investigate the effects of peripheral MS on substance abuse disorders and the role of LH-LHb circuits in the effects, the present study explored whether: (1) peripheral mechanical stimulation (MS) can suppress cocaine-induced locomotor activity, ultrasonic vocalizations (USVs) and NAc DA release, (2) surgical lesions of the LHb suppress the MS effects, (3) MS activates the LHb; (4) optogenetic silencing of the LHb diminishes the MS effects; and (5) chemogenetic inhibition of LH-LHb circuits suppresses the MS-induced inhibition of cocaine-induced locomotion, USVs and drug-seeking behaviors.

## Results

### Effect of LHb lesion on the inhibition by peripheral mechanical stimulation of cocaine-induced 50 kHz USVs and locomotor activity

**Fig. 1:**
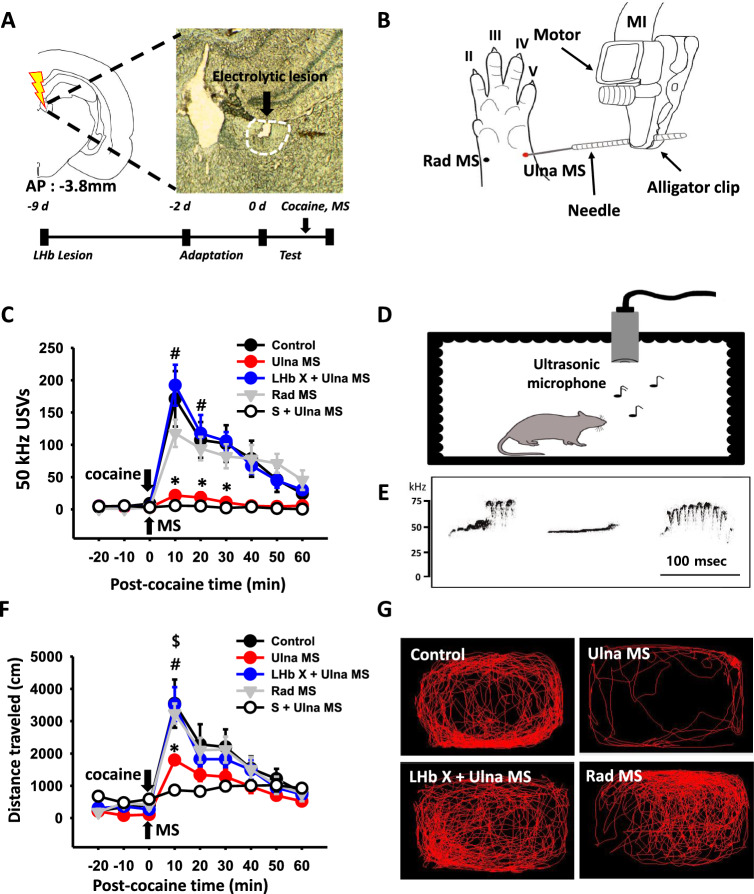
Effect of LHb electrolytic lesions on mechanical stimulation (MS)-induced inhibition of cocaine-enhanced 50 kHz USVs and locomotion. **A** Experiment schedule and electrolytic lesion of LHb stained with toluidine blue. **B** Schematic location of MS sites. The ulnar nerves (Ulna MS) or radial nerves (Rad MS) were stimulated with a mechanical instrument (MI). **C** Effect of MS on cocaine-induced 50 kHz ultrasonic vocalizations (USVs) in normal or LHb-lesioned rats. *p < 0.001, Control versus Ulna MS; ^#^*P* < 0.001, LHb X + Ulna MS versus Ulna MS. n = 7–8 per group. **D**, **E** Schematics for USVs recordings in a sound-attenuating chamber (**D**) and representative spectrograms of 50 kHz USV following cocaine injection (**E**). **F** Effect of MS on cocaine-induced locomotor activity in normal or LHb-lesioned rats. *P < 0.001, Control versus Ulna MS; ^#^*P* < 0.001, LHb + Ulna MS versus Ulna MS; ^$^*P* < 0.01, Ulna MS versus Rad MS. **G** Representative moving tracks for 60 min after cocaine injection or MS (n = 6–7 per group). LHb, lateral habenula; Control, cocaine injection only in normal rats; Ulna MS, mechanical stimulation of ulnar nerve; LHb X + Ulna MS, mechanical stimulation of ulnar nerve after cocaine injection in the rats with electrolytic lesions of bilateral LHb; Rad MS, mechanical stimulation of radial nerve; S + Ulna MS, saline injection instead of cocaine and ulnar MS

To determine whether peripheral mechanical stimulation (MS) can suppress cocaine-induced positive affective states and psychomotor responses, the effects of MS on 50 kHz USVs and locomotor activity following cocaine administration were examined. When cocaine was injected intraperitoneally, the rats emitted 50 kHz USVs of long and high frequencies (Fig. 1d, e) during enhanced locomotion (Fig. 1g). Cocaine significantly increased 50 kHz USVs and locomotor activity, compared to the values before injection, which lasted up to about 60 min with a peak at 10 min (Fig. 1c, f). Cocaine enhancement of 50 kHz USVs and locomotor activity was significantly attenuated by MS (Fig. 1b) applied to ulnar nerve, but not to radial nerve (Fig. 1c, two-way RM ANOVA; treatment, F_(4,160)_ = 12.7688, *P* < 0.001; time, F_(8,160)_ = 22.614, *P* < 0.001; interaction, F_(32,160)_ = 5.878, *P* < 0.001; Fig. 1f, two-way RM ANOVA, treatment, F_(4,160)_ = 7.604, *P* < 0.001; time, F_(8,160)_ = 77.698, *P* < 0.001; interaction, F_(32,160)_ = 3.352, *P* < 0.001). And, ulnar MS itself did not affect locomotor activity and 22 kHz USVs in normal rats (S + Ulna MS in Fig. 1f and Additional file 1: Fig. S1). To test the involvement of LHb in the inhibition of cocaine-enhanced psychomotor responses by ulnar MS, electrolytic lesions of LHb were made bilaterally 7 days before MS treatment (Fig. 1a). Compared to the cocaine + ulnar MS group, ulnar MS failed to inhibit cocaine-enhanced 50 kHz USV and locomotive activity in the rats with LHb lesions (Fig. 1c, two-way RM ANOVA, F_(32,160)_ = 5.878, *P* < 0.001; Fig. 1f, two-way RM ANOVA, F_(32,160)_ = 3.352, *P* < 0.001). LHb lesion itself did not affect basal locomotor activity, compared to normal rats (Additional file 1: Fig. S2A). It demonstrates the involvement of the LHb in the ulnar MS effects on cocaine-induced positive affective states and psychomotor responses. As ulnar MS produced consistent and reproducible inhibitory effects on cocaine-induced locomotion in the present study, MS was applied to the ulnar site in subsequent experiments.

### Effect of LHb lesion on MS-induced inhibition of electrically stimulated dopamine release

**Fig. 2:**
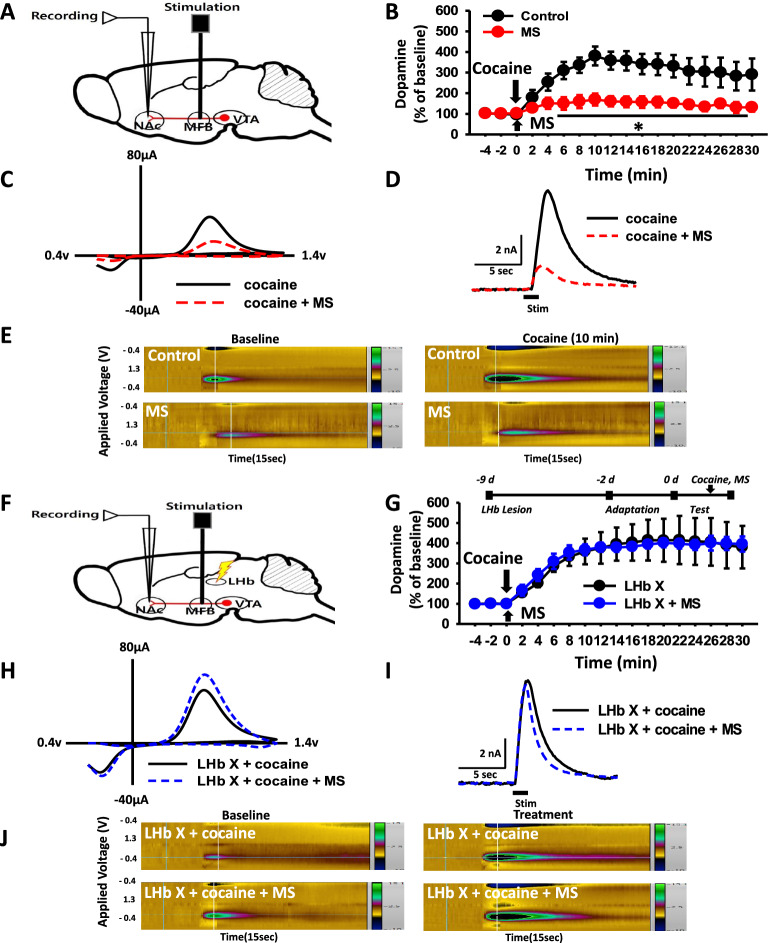
Mediation of LHb in MS inhibition of cocaine-induced DA release in the NAc. **A** Schematic of fast-scan cyclic voltammetry (FSCV) for recording NAc DA release.** B** Effect of MS on cocaine enhancement of electrically stimulated DA release in NAc in normal rats (n = 6 per group). *P < 0.05 versus Control (normal rats). **C–E** Representative voltammograms and representative voltammetry pseudocolor images for each group. **F** Schematic of FSCV for recording NAc DA release in LHb-lesioned rats. **G** Effect of LHb lesions on MS inhibition of electrically stimulated-DA release in NAc (n = 6 per group). **H–J** Representative voltammograms and representative voltammetry pseudocolor images for each group. NAc, nucleus accumbens; DA, dopamine; MFB, medial forebrain bundle; Control, cocaine injection only; cocaine + MS, mechanical stimulation of ulnar nerver in cocaine-injected rats; LHb X, lesion of lateral habenula; LHb X + MS, MS after cocaine injection in LHb-lesioned rats. MS, mechanical stimulation of ulnar nerve

To investigate whether MS suppresses extracellular DA release in the NAc via activation of LHb, in vivo fast-scan cyclic voltammetry (FSCV) was carried out in normal or LHb-lesioned rats (Fig. 2a, f). In normal rats, cocaine injection increased dopamine (DA) release in the NAc by about 400% over baseline, which remained elevated over 30 min after cocaine injection. The increase of DA was attenuated by MS (Fig. 2b, two-way RM ANOVA, treatment, F_(1,150)_ = 107.663, *P* < 0.001; time, F_(14,150)_ = 0.973, *P* = 0.484; interaction, F_(14,150)=_0.439, *P* = 0.960). We repeated the experiments in the rats with bilateral LHb lesions. While cocaine elevated DA release in the NAc in the LHb-lesioned rats, MS did not inhibit DA release in NAc following cocaine injection in LHb-lesioned rats (Fig. 2g). On the other hand, LHb lesion itself did not affect basal DA release in the NAc, compared to normal rats (Additional file 1: Fig. S2B). It indicates the mediation of LHb in the MS effects on cocaine-enhanced NAc DA release.

### Effect of optogenetic silencing of LHb on MS-induced inhibition in cocaine-enhanced 50 kHz USVs and locomotor activity

**Fig. 3:**
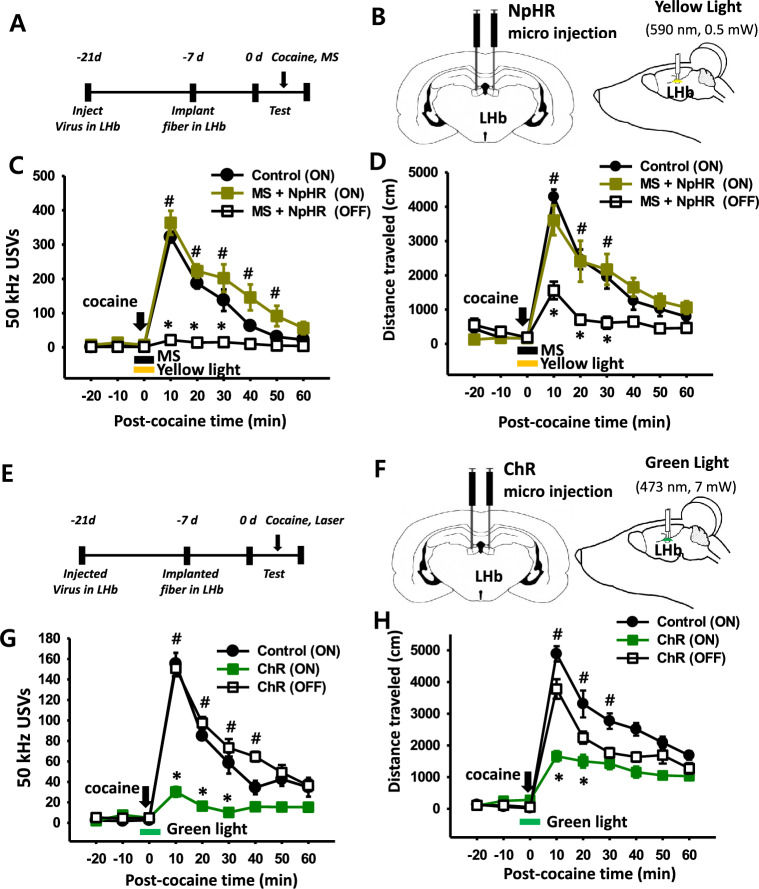
Effect of optogenetic modulation of LHb on MS inhibition of cocaine-induced USVs and locomotion. **A–D** Effect of optogenetic silencing of LHb on MS inhibition of cocaine-induced USVs and locomotion. Experimental schedule (**A**) and schematic for optogenetic inhibition of LHb in rat (**B**). Effect of optogenetic silencing of LHb on MS inhibition of cocaine-induced 50 kHz USVs in rats (**C**). *P < 0.001, Control versus MS + NpHR (OFF); ^#^*P* < 0.001, MS + NpHR (ON) versus MS + NpHR (OFF). Effect of optogenetic silencing of LHb on cocaine-induced locomotor activity in rats (**D**). *P < 0.001, Control versus MS + NpHR (OFF); ^#^*P* < 0.001, MS + NpHR (ON) versus MS + NpHR (OFF). Control (ON, n = 6), switch on yellow light in control virus-injected rats; MS + NpHR (ON, n = 6), switch on of yellow light during MS in NpHR-injected rats; MS + NpHR (OFF, n = 6), switch off of yellow light during MS in NpHR-injected rats. LHb, lateral habenula; MS, mechanical stimulation of ulnar nerve. **E**–**H** Effect of optogenetic activation of LHb on cocaine-induced USVs and locomotion. Experimental schedule (**E**) and schematic for optogenetic activation of LHb in rat (**F**). Effect of optogenetic activation of LHb on cocaine-induced 50 kHz USVs in rats (**G**). *P < 0.001, Control versus ChR (ON); ^#^*P* < 0.001, ChR (ON) versus ChR (OFF). Effect of optogenetic activation of LHb on cocaine-induced locomotor activity in rats (**H**). *P < 0.001, Control versus ChR (ON); ^#^*P* < 0.001, ChR (ON) versus ChR (OFF). Con (ON, n = 6), switch on the green light in control virus-injected rats; ChR (OFF, n = 6), switch off of green light in ChR-injected rat; ChR (ON, n = 6), switch on of green light in ChR-injected rats

To further confirm the mediation of the LHb in MS-induced inhibition of cocaine-elevated 50 kHz USVs and locomotor activity, a viral vector AAV2-hSyn-eNpHR3.0-EYFP (NpHR) was injected into the LHb in order to inhibit LHb regions optogenetically during MS stimulation. Optic fibers were implanted into the LHb 14 days after the virus injection (Fig. 3a, b). In control virus-injected rats, systemic injection of cocaine increased 50 kHz USVs and locomotor activity, lasted up to about 60 min (Fig. 3c, d). MS during yellow OFF-switch inhibited cocaine-induced 50 kHz USVs and locomotor activity, compared to control virus (ON, laser on) group (Fig. 3c, two-way RM ANOVA, treatment, F_(2,96)_ = 46.648, *P* < 0.001; time, F_(8,96)_ = 71.422, *P* < 0.001; interaction, F_(16,96)_ = 14.390, *P* < 0.001; Fig. 3d, two-way RM ANOVA, treatment, F_(2,96)_ = 11.028, *P* = 0.002; time, F_(8,96)_ = 50.670, *P* < 0.001; interaction F_(16,96)_ = 7.014, *P* < 0.001; MS + NpHR (OFF) vs. MS + NpHR (ON) + Control (ON), *^,#^*P* < 0.001). When mechanical stimulation during the yellow ON-switch was performed (Fig. 3b), the inhibitory effects of MS on cocaine-enhanced behaviors were attenuated (Fig. 3c, d).

### Effect of optogenetic activation of LHb in cocaine-induced 50 kHz USVs and locomotor activity

To evaluate whether LHb activation itself can suppress cocaine-induced psychomotor responses, a viral vector AAV2-hSyn-ChR2 (E123A)-EYFP (ChR) was implanted into LHb to activate the LHb region optogenetically (Fig. 3e, f). In control virus-injected rats, systemic injection of cocaine during emission of green light increased 50 kHz USVs and locomotor activity (Fig. 3g, h). On the other hand, in ChR (ON) group, optogenetic activation of LHb by green light emission significantly inhibited cocaine-induced 50 kHz USVs and locomotor activity, compared to control or ChR (OFF) group (Fig. 3g; treatment, F_(2,80)_ = 57.901, *P* < 0.001; time, F_(8,80)_ = 65.175, *P* < 0.001; interaction F_(16,80)_ = 11.001, *P* < 0.001; Fig. 3h, two-way RM ANOVA, treatment, F_(2,80)_ = 6.538, *P* = 0.015; time, F_(8,80)_ = 92.032, *P* < 0.001; interaction, F_(16,80)_ = 5.344, *P* < 0.001).

### Activation of LHb neurons by MS in acute cocaine-treated rats

**Fig. 4:**
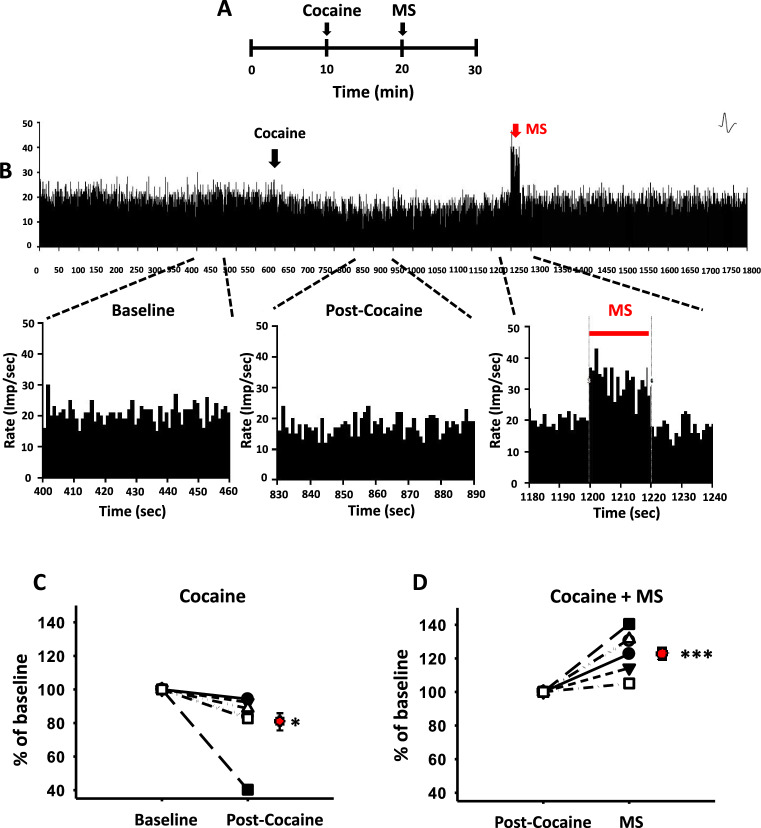
In vivo extracellular single-unit recordings of LHb neurons during mechanical stimulation. **A** Experimental schedule. **B** Single-unit discharges from LHb neurons for total 30 min. **C** Individual percent change of spike frequency/sec before (Baseline) and after cocaine (Post-Cocaine) and the mean percent change (red filled circle). **D** Individual percent change of spike frequency/sec before (Post-Cocaine) and after MS (MS) and the mean percent change (red filled circle). Mean spike frequency/sec before and after cocaine and/or MS. Single-unit activities of LHb neurons were evoked during MS in cocaine-injected rats. *P < 0.001. MS, mechanical stimulation of ulnar nerve. n = 6

To determine whether MS excites LHb neurons, in vivo extracellular single-unit recordings were performed in LHb neurons during mechanical stimulation of ulnar nerve. After a stable baseline for at least 10 min was established, neuronal activity following cocaine injection and MS treatment was recorded for another 20 min (Fig. 4a, b). Systemic injection of cocaine (15 mg/kg, i.p.) decreased the firing rate of LHb neurons to 80.39 ± 5.55% over baseline value within 10 min after injection (Fig. 4b, c; One-way RM ANOVA, F_(1,10)_ = 5.674, *P* < 0.05, Baseline vs. Post-Cocaine). When MS was applied 10 min after cocaine injection, single-unit discharges were increased 124.08 ± 3.53% over baseline (Fig. 4b, d; One-way RM ANOVA, F_(1,10)_ = 21.186, *P* < 0.001, Post-Cocaine vs. MS).

### Effect of chemogenetic inhibition of LH-LHb circuit on MS inhibition of cocaine-induced 50 kHz USVs and locomotor activity

**Fig. 5:**
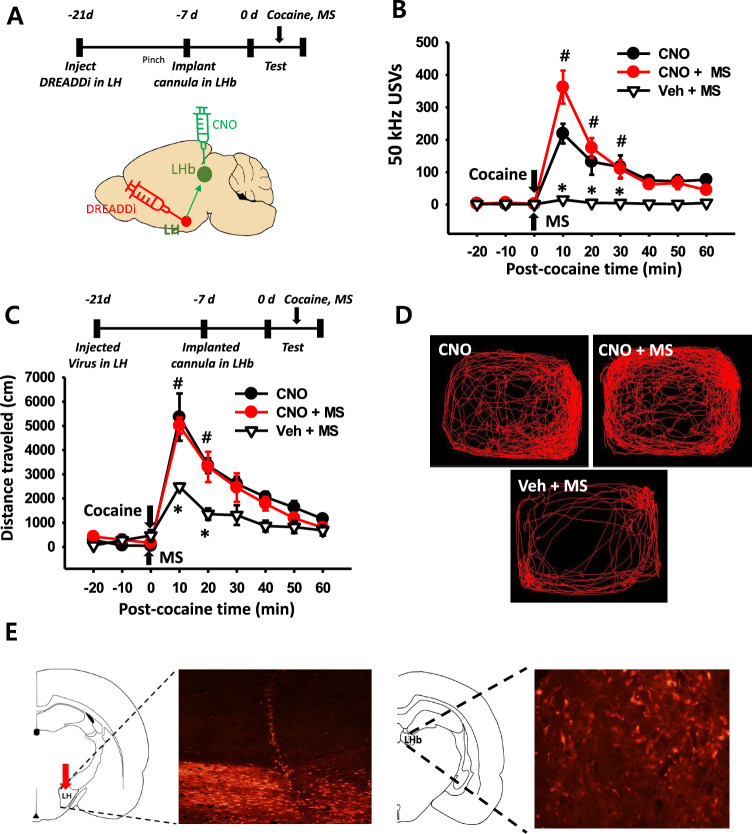
Effect of LH-LHb inhibition with DREADDi on cocaine-induced 50 kHz USVs and locomotion. **A** Experimental schedule and schematics for chemogenetic inhibition of LH-LHb pathway. **B** Effect of LH-LHb inhibition with DREADDi on cocaine-induced 50 kHz USVs. *P < 0.001 CNO versus Veh + MS; ^#^*P* < 0.001, CNO + MS versus Veh + MS. n = 6 per group. **C**,** D** Effect of LH-LHb inhibition with DREADDi on cocaine-induced locomotor activity. *P < 0.001 CNO versus Veh + MS; ^#^*P* < 0.001, CNO + MS versus Veh + MS. Representative moving traces for 60 min after treatments of cocaine, CNO or CNO + MS; CNO + MS, CNO injection before MS in cocaine-injected rats; Veh + MS, aCSF injection before MS in cocaine-injected rats. **E** Viral DREADDi expression in LH or LHb. MS, mechanical stimulation of ulnar nerve. n = 6 per group

To determine the role of the LH-LHb circuit in mediating the MS effects on cocaine-induced 50 kHz USVs and locomotor activity, the LH-LHb projection was inhibited using DREADD-based chemogenetics during MS treatment. The DREADD-encoding virus was injected into LH and a cannula was implanted into LHb (Fig. 5e). CNO was then injected into LHb for chemogenetics inhibition of the LH-LHb (Fig. 5a, e). MS significantly inhibited cocaine-enhanced 50 kHz USVs and locomotor activity, compared to the CNO group (Veh + MS in Fig. 5b–d, two-way RM ANOVA, F_(16,161)_ = 9.448, *P* < 0.001; Fig. 5c, two-way RM ANOVA, F_(16,80)_ = 4.615, *P* < 0.001). When CNO was infused into LHb prior to MS treatment, the inhibitory effects of MS on cocaine-induced behaviors were almost completely ablated, compared to CNO group (CNO + MS group in Fig. 5b, two-way RM ANOVA, F_(16,161)_ = 9.448, *P* < 0.001; Fig. 5c; two-way RM ANOVA, F_(16,80)_ = 4.615, *P* < 0.001).

### Effect of inhibition of LH-LHb circuit with DREADD on MS-induced suppression of the reinstatement of cocaine-seeking behavior

**Fig. 6:**
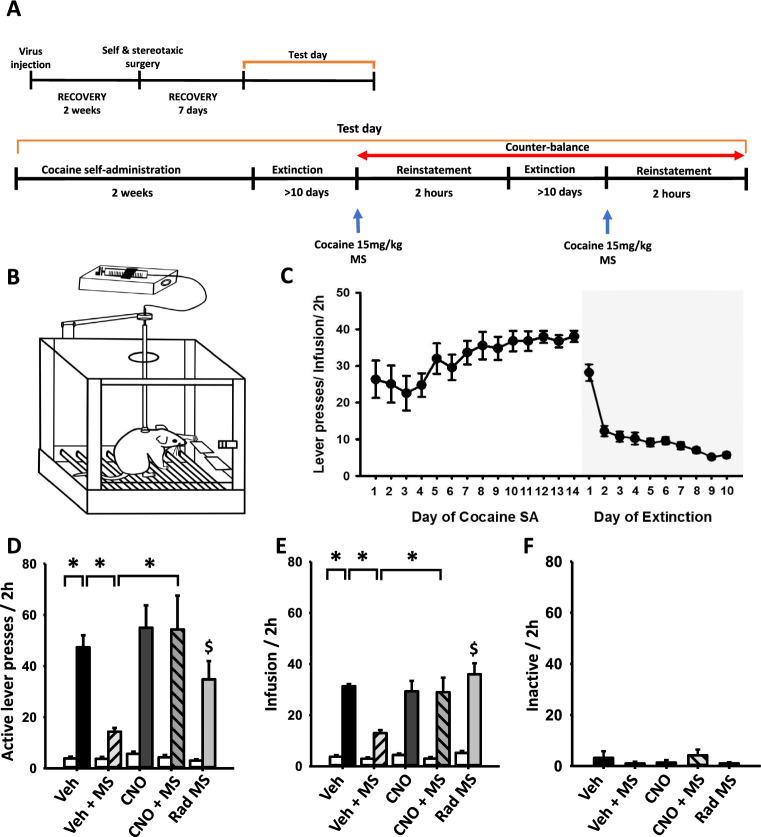
Effect of chemogenetic inhibition of LH-LHb on MS inhibition of cocaine-induced reinstatement. **A** Experimental schedule for cocaine self-administration. **B** Schematic of cocaine self-administration procedure in rats. **C** The number of lever presses during cocaine self-administration and extinction training (n = 6). **D**–**F** Effect of chemogenetic inhibition of LH-LHb on MS suppression of cocaine priming-induced reinstatement of drug seeking. The number of total active lever (**D**), infusion (**E**) and inactive lever (**F**) pressings during the reinstatement test session (2-h) following a priming injection of cocaine (15 mg/kg, i.p.) in rats. CNO, CNO administration in the rats given cocaine-primed injection; CNO + MS, CNO administration during MS in the rats given cocaine-primed injection; Veh + MS, aCSF infusion during MS in the rats given cocaine-primed injection. *P < 0.001; ^$^*P* < 0.05, Veh + MS versus Rad MS. White bar in each group represents the number of lever responding before cocaine-primed injection. MS, mechanical stimulation of ulnar nerve; Rad MS, mechanical stimulation of radial nerve. n = 6 per group

The groups were assigned for cocaine-seeking behavioral experiments as follows: (1) Veh (vehicle, n = 6), vehicle (aCSF) injection instead of CNO before cocaine-primed reinstatement test; (2) Veh + MS (n = 6), vehicle injection + mechanical stimulation of ulnar nerve before cocaine-primed reinstatement test; (3) CNO (n = 6), CNO injection before cocaine-primed reinstatement test; and (4) CNO + MS (n = 6), CNO injection + MS in cocaine-primed reinstatement test (Fig. 6a, b). Rats had acquired cocaine self-administration over 14 days and presented stable behavioral responses on lever presses during the last 5 days (Fig. 6c). During extinction sessions, the rats showed decay of active lever responding across sessions and their active lever presses were reduced to < 10% of the established baseline from the initial extinction session (Fig. 6c). When cocaine was challenged, the rats exhibited significant increases in responses of the active lever or infusions (42.8 ± 7; Veh group in Fig. 6d, e), but not inactive lever (Veh group in Fig. 6f), compared to the corresponding values before cocaine-priming injection (25.7 ± 3; white bars in Fig. 6d, e). To test whether MS might attenuate cocaine-induced reinstatement of cocaine-seeking, the rats received ulnar MS after cocaine injection and then lever presses were recorded for 2 h. MS significantly reduced cocaine-induced reinstatement, compared to the control group (Veh, *P* < 0.05; Fig. 6d, e), while MS did not affect the responses of inactive lever (Fig. 6f). To inhibit the LH-LHb circuit during MS treatment, CNO was infused into LHb in DREADDi-injected rats and MS was performed. Injection of CNO into LHb prior to MS blocked the inhibitory effects of MS on cocaine-induced active lever presses (Fig. 6d; one-way RM ANOVA, F_(9,45)_ = 15.246, *P* < 0.001) and infusions (Fig. 6e; one-way RM ANOVA, F_(9,45)_ = 26.242, *P* < 0.001).

### Effect of optogenetic inhibition or activation during MS on c-fos expression in the LHb

**Fig. 7:**
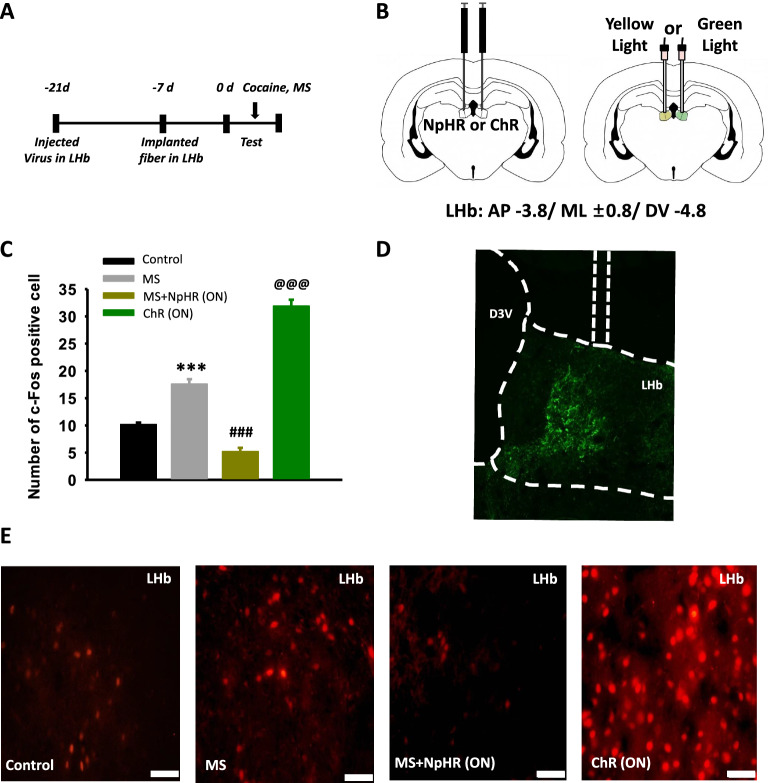
Effects of mechanical stimulation and optogenetic inhibition on c-Fos expression in the LHb. **A** Experimental schedule. **B** Schematic for virus injection and optogenetics in LHb.** C** Mean numbers of the c-Fos positive neurons in the LHb in each group. ***P < 0.001, Control versus MS; ^###^*P* < 0.001, MS versus MS + NpHR (ON); ^@@@^*P* < 0.001, MS + NpHR (ON) versus ChR (ON). **D** Verification of virus injection site on the end of experiment. **E** Representative image of c-Fos expression in the LHb in control group (n = 6), MS group (n = 6), MS + NpHR (ON) group (n = 6), and ChR (ON) group (n = 6). Bar = 50 µm. MS, mechanical stimulation of ulnar nerve. ON, switch on the laser light. OFF, switch off the laser light

Finally, to verify whether MS activates LHb neurons or optogenetic inhibition during MS silences LHb neurons, another set of animals (n = 6 per group) was divided into the following groups: (1) Control, naïve rats; (2) MS, mechanical stimulation during light emission of LHb in control virus-injected rats; (3) MS + NpHR (ON), mechanical stimulation during light emission of LHb in NpHR virus-injected rats: and (4) ChR, light emission of LHb in ChR virus-injected rats. c-Fos expression was evaluated in the LHb (Fig. 7a, b). While MS increased c-Fos expression in the LHb, the increase was inhibited in the rats given yellow light with MS treatment (Fig. 7c–e, one-way ANOVA, F_(3,23)_ = 194.397, *P* < 0.001). Green light of LHb increased c-Fos expression in ChR-injected rats. (Fig. 7c, one-way ANOVA, F_(3,23)_ = 194.397, *P* < 0.001).

## Discussion

In the present study, mechanical stimulation (MS) applied to ulnar nerve attenuated cocaine enhancement of 50 kHz USVs, locomotor activity and NAc DA release, which was blunted by chemical lesion of LHb. MS inhibition of cocaine-induced psychomotor responses was blocked by optogenetic inhibition of LHb during MS. Optogenetic stimulation of the LHb reduced the cocaine psychomotor responses. MS excited LHb neurons directly in cocaine-treated rats. The MS effects on cocaine-enhanced 50 kHz USVs and locomotion was suppressed by chemogenetic inhibition of the LH-LHb circuit. MS also reduced cocaine-induced reinstatement of drug seeking behaviors, which was blocked by chemogenetic silencing of the LH-LHb circuit. These findings suggest that peripheral MS recruits the LH-LHb circuit to reduce cocaine addiction-like behaviors.

The present study showed that MS of ulnar nerve, but not radial nerve, attenuated cocaine-enhanced locomotor behaviors and cocaine-priming reinstatement of drug seeking behaviors. Radial stimulation was applied, as the corresponding control site to ulnar MS, on the opposite side of the forelimb, about 5 mm apart from site of ulnar MS. Radial MS did not affect cocaine-induced behaviors, cocaine-primed active lever presses or 22 kHz USVs, an indicator of pain or discomfort [11]. These findings suggest that reduction of cocaine-induced behaviors by ulnar MS was not due to wrist pain from MS. It is consistent with previous studies that mechanical stimulation of ulnar nerve at HT7, an acupoint at the ulnar nerve, suppressed cocaine-induced locomotor activity [3], cocaine-seeking behavior [12], morphine self-administration behavior or ethanol self-administration behavior [13]. During such stimulation, the superficial or deep afferents of ulnar nerve are activated and the afferent signals are transmitted via A-fiber of ulnar nerve [4]. The afferent inputs reduce drug-induced DA release in the NAc by modulating VTA GABA neurons in addicted rats [2, 3, 14]. It suggests that mechanical stimulation may reduce drug addictive behavior in a nerve-dependent manner.

The present study showed that peripheral mechanical stimulation suppressed cocaine enhancement of 50 kHz USVs and NAc DA release. It is known that 50 kHz USV occur during a positive affective state such as rewarding events, play behavior, mating and addictive drugs and the 50 kHz USV is associated with NAc DA release [15]. Our previous studies have shown that peripheral mechanical stimulation modulates GABA neuron firing rates in the VTA, DA release in the NAc [2, 12]. These findings suggest that mechanical stimulation of ulnar nerve attenuates cocaine-enhanced psychomotor activity by inhibiting mesolimbic DA system.

The present study showed that attenuation by MS of cocaine-induced psychomotor responses was ablated by disruption of LHb. Optogenetic inhibition of LHb during MS blocked an inhibitory effect of MS on cocaine-induced psychomotor responses (50 kHz USV and locomotion). Optogenetic stimulation of LHb itself suppressed the cocaine behaviors. MS excited LHb neurons in cocaine-treated rats. Our findings may indicate that peripheral MS recruits LHb to suppress cocaine-induced psychomotor responses. Cumulative evidence has suggested that LHb is a critical epithalamic structure interconnecting sensory inputs to mesolimbic DA systems [16]. The LHb participates in the processing of nociceptive stimulation [17, 18]. Neurons in the LHb directly innervate midbrain rostromedial tegmental nucleus (RMTg) GABA neurons, and indirectly inhibit DA neurons [8]. It has been accepted that the LHb neurons convey aversive and negative reward conditions through potent inhibition of VTA DA neurons and NAc DA release [19]. Previous studies showed that electrical activation of LHb reduces cocaine-seeking behavior [20] but inhibition of LHb increases cocaine seeking [21]. Therefore, we suggest that MS activates LHb neurons and leads to activation of RMTg GABA neurons and subsequent inhibition of VTA DA neurons that result in suppression of cocaine-enhanced psychomotor responses. Yang et al. showed that electrical stimulation of the HT7 acupuncture point, over ulnar nerve, inhibits VTA GABA neuron activity with recovery in 5 min in normal rats but increases the activity of VTA GABA neuron activity suppressed by acute alcohol administration in rats [14]. Our previous study also showed that mechanical stimulation of HT7 increases the VTA GABA neuron activity suppressed by acute cocaine administration in rats [12]. Taken together, we suggest that ulnar MS would recover cocaine suppression of VTA GABA neuron activity and thus decrease acute cocaine-induced DA release in the NAc and psychomotor responses.

In the present study, chemogenetic silencing of a LH-LHb circuit suppressed the inhibitory effect of MS on cocaine-enhanced 50 kHz USV and locomotor behaviors. Furthermore, MS reduced cocaine-priming reinstatement of drug seeking behaviors, which was ablated by chemogenetic inhibition of an LH-LHb circuit. These data suggest that MS reduces cocaine-induced addictive behaviors via an LH-LHb circuit. As shown in our present and previous tracing studies [3], the LH innervates the LHb that in turn is connected to the RMTg GABA neurons and VTA DA system [22]. Previous studies have shown that the LH is involved in drug-taking behaviors, reinstatement of drug-seeking behavior, and drug-induced synaptic plasticity [23, 24]. It was reported that degradation of the LH structures inhibits cocaine cue-induced reinstatement of drug-seeking behavior in rats [24]. The LHb also plays a critical role in drug-induced craving and aversion [25]. The LH-LHb pathway encodes negative valence and controls motivational behaviors [26, 27]. Stimulation of the LH-LHb pathway inhibits the mesolimbic DA system and reward-related behaviors [8, 27]. Therefore, we suggest that MS activates the LH-LHb circuit, which in turn activate RMTg GABA neurons and suppress mesolimbic DA system, thereby leading to reduction of cocaine addictive behaviors.

## Conclusion

These findings suggest that mechanical stimulation of ulnar nerve activates LH-LHb pathway and thus attenuates cocaine-induced psychomotor responses and drug seeking behaviors.

## Methods

### Animals

All experiments were performed with male Sprague–Dawley rats (Hyochang, Seoul, Korea), weighing 210–400 g. The animals were housed under a constant temperature (24 ± 2 °C) and a 12-h light–dark cycle with free access to food and water. All procedures were approved by the Institutional Animal Care and Use Committee (IACUC) at Daegu Haany University and conducted according to National Institutes of Health Guide for Care and Use of Laboratory Animals. Total 180 rats were used and 10 rats were excluded due to technical failures of LHb lesion (n = 2) and loss of jugular vein catheters during cocaine self-administration experiment (n = 8).

### Chemicals and reagents

Cocaine (15 mg/kg in saline; Macfarlan Smith Ltd, UK), AAV2-hSyn-EYFP (control virus; viral titer ≥ 7 × 10^12^ vg/mL; 0.5 μL/loci), adeno-associated virus-2 vectors driven by human alpha-synuclein promoter expressing halorhodopsin (AAV2-hSyn-eNpHR3.0-EYFP, NPHR; viral titer ≥ 7 × 10^12^ vg/mL; 0.5 μL/loci) or channelrhodopsin (AAV2-hSyn-ChR2 (E123A)-EYFP, ChR; UNC viral vector core, NC, USA; viral titer ≥ 7 × 10^12^ vg/mL; 0.5 μL/loci), adeno-associated virus vectors (pAAV-hSyn-hM4D(Gi)-mCherry, Addgene, Watertown, MA, USA; viral titer ≥ 10^12^ vg/mL; 0.5 μL/loci) for inhibitory designer receptors exclusively-activated by designer drugs (DREADDi), and clozapine N-oxide dihydrochoride (CNO, Tocris Bioscience, Bristol, UK) were used.

### Mechanical stimulation of peripheral nerve

Peripheral mechanical stimulation was carried out by using a mechanical instruments (MI) developed in our laboratory [3], as described in our previous experiment [3]. In brief, for mechanical stimulation of ulnar nerve (Ulnar MS), needles (0.18 mm in diameter, 8 mm in length, DongBang Medical Co., Qingdao, China) were inserted bilaterally 3 mm deep into the ulnar tunnel on the transverse crease of the wrist of the forepaw. By using our MI that consisted of a custom-made control unit and a mechanical vibrator connected to the needle, the inserted needle was mechanically stimulated for 20 s in duration at an intensity of 1.3 m/s^2^, maintained for 1 min after needle insertion and subsequently withdrawn. To assess the possibility that the mechanical stimulation of the wrist impaired locomotor behaviors, the radial nerve side of wrist joint was mechanically stimulated in an identical fashion as a control condition (Radial MS). Immediately after cocaine injection, MS was applied for 20 s.

### Measurement of locomotor activity and USVs

Locomotor activity and USVs were recorded simultaneously in customized sound-attenuating chambers. The chamber consisted of two boxes to minimize exterior noise (inside box: 54 × 38 × 35 cm, outside box: 68 × 50 × 51 cm). A condenser ultrasonic microphone (Ultramic250K; Dodotronic, Castel Gandolfo, Italy) and a digital camera were positioned at the center of the ceiling of the chamber. As performed in our laboratory [28], 50 kHz USVs were recorded using the ultrasonic microphone with UltraSoundGate 416H data acquisition device (Avisoft Bioacoustics, Glienicke, Germany). Ultrasonic vocal signals were band-filtered between 38 and 96-kHz for the 50 kHz USVs and 18–26 kHz for the 22 kHz USVs, respectively, and analyzed using Avisoft-SASLab Pro (version 4.2, Avisoft Bioacoustics). Locomotor activity was measured with a video-tracking system (Ethovision XT; Noldus Information Technology BV, Wageningen, Netherlands). After recording baseline activity for 30 min, the rats were given an intraperitoneal (i.p.) injection of cocaine (15 mg/kg) and/or mechanical stimulation and monitored up to 60 min after cocaine injection. The data were expressed as the distance traveled for locomotor behaviors and numbers of USVs for vocalizations during each 10-min period, respectively.

### Fast-scan cyclic voltammetry (FSCV)

Electrically evoked-DA release in the NAc was measured by fast-scan cyclic voltammetry (FSCV) in vivo, as described previously [29, 30]. Briefly, a 7.0-μm diameter carbon fiber was inserted into borosilicate glass capillary tubing (1.2 mm o.d., A-M Systems, WA, USA) under negative pressure and subsequently pulled on a vertical pipette puller (Sutter Instrument, Model P-97, Nocato, CA, USA). The carbon fiber electrode (CFE) was then cut under microscopic control with 200-μm of bare fiber protruding from the end of the glass micropipette. The tip of the CFE was sealed using cyanoacrylate and back-filled with 3 M KCl. The electrode potential was scanned with a triangular waveform from − 0.41.3 V and back to − 0.4 V versus Ag/AgCl at a scan rate of 400 V/second. Cyclic voltammograms were recorded every 100 ms (i.e. 10 Hz) by a ChemClamp voltage clamp amplifier (Dagan Corporation, Minneapolis, MN, USA). The FSCV recordings were performed and analyzed using LabVIEW-based (National Instruments, Austin, TX, USA) customized software (Demon Voltammetry) [31]. For in vivo FSCV recordings of DA signals, rats were anesthetized with urethane (1.5 g/kg, i.p.) and placed in a stereotaxic apparatus (David Kopf Instruments, Tujunga, CA, USA). The body temperature was maintained constant at 37 °C using a feedback-controlled DC heating pad. A bipolar, coated stainless steel electrode was stereotaxically implanted into the medial forebrain bundle (MFB: AP − 2.5 mm, ML + 1.9 mm, DV − 8.0 to  − 8.5 mm from the skull) and CFE in the NAc (AP + 1.6 mm, ML + 1.9 mm, DV − 6.5 to  − 8.0 mm). The MFB was stimulated with 60 monophasic pulses at 60 Hz (4-ms pulse width) and received continuous stimulation at intervals of 2 min. Once the stimulated DA response was stable for three successive collections, and did not differ by more than 10%, baseline measurements were taken for mechanical stimulation and drug treatment. After a stable baseline was established, rats received an injection of cocaine (15 mg/kg, i.p.) and/or mechanical stimulation. The DA signals were recorded at 2 min-intervals for 30 min after cocaine injection.

### Electrolytic lesions of lateral habenula (LHb)

Electrolytic lesions of LHb were made as described previously [3]. Briefly, under sodium pentobarbital anesthesia (50 mg/kg i.p.) the rat was placed on the stereotaxic apparatus and small holes were made in the skull bilaterally over the LHb region. A tungsten electrode, insulated except for 0.5 mm at the tip, was inserted in LHb (AP − 3.72 mm, ML ± 0.8 mm, DV − 4.9 mm) [32]. The bilateral electrolytic lesions of LHb were made by passing ± 0.35 mA positive current for 8 s. The rats were allowed to recover for a week prior to experimentation.

### Histological examination of lesion

At the end of the experiments, all rats were sacrificed for histological confirmation of the lesions. All animals were perfused with 0.9% NaCl and then with 4% paraformaldehyde (PFA). The brains were removed, post-fixed in 4% PFA and cryoprotected in 30% sucrose. The brain tissues were cryosectioned 30 μm-thick and dyed with toluidine blue. Only those rats with correctly placed lesion were included for data analysis.

### Optogenetic inhibition or activation of LHb

Under pentobarbital anesthesia (50 mg/kg, i.p.) viral vectors, AAV2-hSyn-EYFP (control virus), AAV2-hSyn-eNpHR3.0-EYFP (NpHR) or AAV2-hSyn-ChR2 (E123A)-EYFP (ChR) were injected into bilateral LHb (AP − 3.8 mm, ML ± 0.8 mm, DV − 4.8 mm) at the rate of 0.25 μL/mL using a Hamilton syringe (26-gauge, USA) connected with a microinjection pump (pump 22, Harvard Apparatus, MA, USV). Two weeks after injection, optic fibers were implanted to target the LHb. The rats were then allowed to recover for 1 week prior to experimentation. For optogenetic inhibition or activation of LHb neurons, mating ferrules were connected to the implanted optic fibers and illuminated for 60 s at 590 (constant 0.5 mW yellow light for NpHR) or 473 nm (7 mw green light, 20 Hz, 10 ms ON for ChR) by using an optogenetic system (Doric Lenses, Quebec, Canada). At the end of experiments, the rats were sacrificed for histological confirmation of injection sites and viral expression.

### In vivo extracellular recording of LHb neurons

Single-unit discharges from LHb neurons were recorded as described previously [3, 16]. Briefly, under urethane anesthesia (1.5 g/kg, i.p.), rats were positioned on a stereotaxic apparatus and small holes were drilled into the skull to accommodate recording electrodes. A single carbon-filament glass microelectrode (Carbostar-1, impedance 0.4–1.2 MΩ, Kation Scientific, USA) were stereotaxically positioned in the LHb area (AP − 3.6 to  − 3.8 mm, ML ± 0.8 to  ± 0.9 mm, DV − 4.8 to  − 5.4 mm from the skull) [32]. After a stable baseline of at least 10 min was established, the unit activity was recorded for another 20 min by simulating cocaine and mechanical stimulation.

### Chemogenetics and cocaine self-administration

We adopted a DREADDi-based chemogenetic tool for selective inhibition of the LH–LHb circuit as described previously [26]. Briefly, animals were anesthetized with pentobarbital sodium (50 mg/kg, i.p.) and a DREADDi-encoding virus (pAAV-hSyn-hM4Di(Gi)-mCherry) was injected into the LH region (AP − 2.5 mm, ML ± 1.4 mm, DV − 8.8 mm) using a 10 μL Hamilton syringe (Hamiton Americas & Pacific Rim, USA) controlled by a syringe pump at a rate of 0.25 μL/6 min. Two weeks after injecting the viral vectors, a double-barreled guide cannula (26-gauge, Double 1.6 mm C–C, USA) was implanted in the bilateral LHb.

Cocaine self-administration procedures were carried out in operant chambers (Med Associates, St. Albans, VT, USA) equipped on wall with active levers, inactive levers, a white house light, and a stimulus light, as described previously [29]. Two weeks after the DREADDi-encoding virus was injected into LH, the rats were implanted with double-barreled cannulas into the bilateral LHb, subjected to a catheter surgery (Dow Corning, Midland, MI, USA) in the right jugular vein and allowed to recover for at least 7 days before training. The rats were trained to self-administer cocaine during a 2-h daily session under FR1 schedule over 2–3 weeks. Each response on the active lever delivered cocaine intravenously (0.5 mg/kg in 0.1 mL over 5 s) and provided an additional 10 s "time-out" period (TO) following a cue light located on the active lever, but the inactive lever gave nothing. Following the establishment of the stable responses, baseline levels of responding were defined as the average value in three consecutive sessions with less than 10% variation. After the establishment of stable cocaine self-administration, the rat underwent 2-h daily extinction sessions for a minimum of 10 days. During extinction sessions, the same conditions as those of the cocaine self-administration were maintained, except that cocaine was replaced with saline. Once the rats met the extinction criteria (below 10% of the responses at the active lever reached during maintenance over at least 3 consecutive days), the rats were tested for the response reinstatement induced by a self-administered reinforcement (15 mg/kg cocaine, i.p.). Prior to a priming injection of cocaine, the rats were given either vehicle or vehicle + MS. The rats were then subjected to 2-h daily extinction sessions for a minimum of 10 days. A within-subjects design was used in which each rat received either vehicle or vehicle + MS in a counterbalanced order across two sessions. In another set of animals, they received the above procedures of cocaine self-administration and the two extinctions and tested for the effects of either CNO or CNO + MS on cocaine-induced reinstatement in a counterbalanced order. The CNO was injected 2 min before MS through a double-barreled cannula implanted in the bilateral LHb.

### Immunohistochemistry for c-Fos expression

Rats were treated with cocaine and MS 30 min before sacrificed. The brains were perfused with 0.9% NaCl and 4% paraformaldehyde (PFA), post-fixed, cryoprotected in 30% sucrose and cryosectioned into 30-μm thick. The sections were incubated with anti-c-Fos rabbit polyclonal antibodies (1:2000; ab 190,289) followed by an incubation of secondary antibody (Donkey anti-rabbit Alexa Fluor 594, red; 1:500) for 2-h at room temperature and mounted onto gelatin-coated slides. The slices were photographed and examined under a confocal laser scanning microscope (LSM700, Carl Zeiss, Germany). The number of c-Fos was blindly counted in bilateral LHb areas. The 4–6 brain pieces per animal were analyzed and averaged.

### Statistical analyses

Data were presented as the mean ± SEM (standard error of the mean) and analyzed by one- or two-way repeated measurement (RM) analysis of variance (ANOVA), followed by post hoc testing using the Tukey method or paired t-test, where appropriate. Values of *P* < 0.05 were regarded as statistically significant.

## Supplementary Information

**Additional file 1. Fig. S1**: Effect of MS on 22 kHz ultrasonic vocalizationsin saine-treated rats. **A**, **B** Changes of 22 kHz USVs following ulnar mechanical stimulationand a representative 22 kHz USV. **Fig. S2**: Effect of electrolytic LHb lesion on locomotor activity or basal DA release in the NAc in rats. **A** Effect of electrolytic LHb lesion on basal locomotor activity. Seven days after LHb lesion, locomotor activities were measured for 30 min. There are no significant differences in basal locomotor activities between normaland LHb-lesionedrats. **B** Effect of electrolytic LHb lesion on basal dopamine release. Seven days after LHb lesion, FSCV experiments were performed after locomotor activity tests. There are no significant differences in basal dopamine release in the NAc between normaland LHb-lesionedrats.

## Data Availability

All data generated or analysed during this study are included in this published article.
