# Supplementary figures and images for: Activation of a hypothalamus-habenula circuit by mechanical stimulation inhibits cocaine addiction-like behaviors
Source: Biol Res. 2023 May 17;56:25. doi: 10.1186/s40659-023-00440-7 (PMC10190066; doi:10.1186/s40659-023-00440-7)

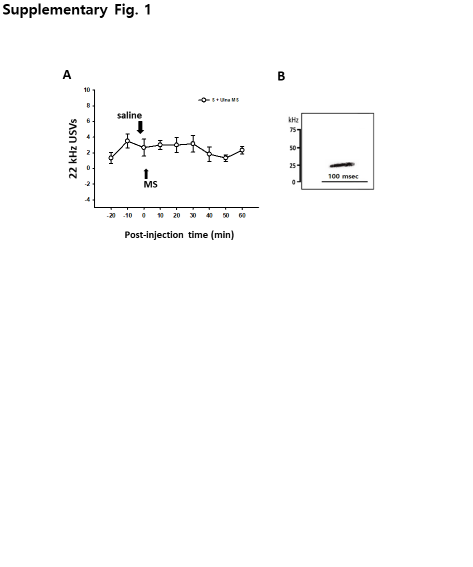


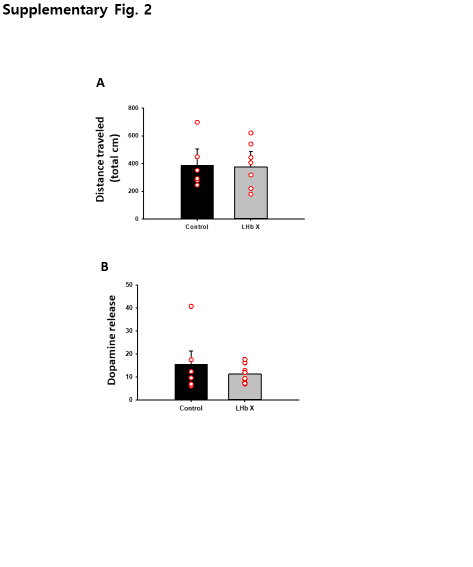

Supplement: Supplementary file 1 — Additional file 1. Fig. S1: Effect of MS on 22 kHz ultrasonic vocalizationsin saine-treated rats. A, B Changes of 22 kHz USVs following ulnar mechanical stimulationand a representative 22 kHz USV. Fig. S2: Effect of electrolytic LHb lesion on locomotor activity or basal DA release in the NAc in rats. A Effect of electrolytic LHb lesion on basal locomotor activity. Seven days after LHb lesion, locomotor activities were measured for 30 min. There are no significant differences in basal locomotor activities between normaland LHb-lesionedrats. B Effect of electrolytic LHb lesion on basal dopamine release. Seven days after LHb lesion, FSCV experiments were performed after locomotor activity tests. There are no significant differences in basal dopamine release in the NAc between normaland LHb-lesionedrats. [file 40659_2023_440_MOESM1_ESM.docx]
